# Toxigenic *Corynebacterium ulcerans* isolated from a wild bird (ural owl) and its feed (shrew-moles): comparison of molecular types with human isolates

**DOI:** 10.1186/s13104-016-1979-5

**Published:** 2016-03-22

**Authors:** Chihiro Katsukawa, Kaoru Umeda, Ikuko Inamori, Yuka Kosono, Tomokazu Tanigawa, Takako Komiya, Masaaki Iwaki, Akihiko Yamamoto, Susumu Nakatsu

**Affiliations:** Department of Infectious Diseases, Osaka Prefectural Institute of Public Health, 1-3-69 Nakamichi, Higashinari-ku, Osaka-Shi, Osaka 537-0025 Japan; Department of Microbiology, Osaka City Institute of Public Health and Environmental Sciences, 8-34 Tojo-cho, Tennoji-ku, Osaka-Shi, Osaka 543-0026 Japan; Nature Conservation Club of Soenji, 1-20-11 Himurodai, Hirakata-Shi, Osaka 573-0115 Japan; Bird Bander, 5-17 Suganodai, Nara-Shi, Nara 631-0043 Japan; Wild Living Thing Society of Hirakata, 1-24-10, Tanokuchiyama, Hirakata-Shi, Osaka 573-0001 Japan; Department of Bacteriology II, National Institute of Infectious Diseases, 4-Gakuen, Musashimurayama-Shi, Tokyo 208-0011 Japan; Nakatsu Animal Hospital, 2-2-15 Shorinji-cho nishi, Sakai-ku, Sakai-Shi, Osaka 590-0960 Japan; Division of Biosafety Control and Research, National Institute of Infectious Diseases, 4-7-1 Gakuen, Musashimurayama-Shi, Tokyo 208-0011 Japan

**Keywords:** Zoonosis, Diphtheria, Food chain, Wildlife, Infection source

## Abstract

**Background:**

*Corynebacterium ulcerans* is a pathogen causing diphtheria-like illness to humans. In contrast to diphtheria by *Corynebacterium diphtheriae* circulating mostly among humans, *C. ulcerans* infection is zoonotic. The present study aimed to clarify how a zoonotic pathogen *C. ulcerans* circulates among wild birds and animals.

**Results:**

By screening 380 birds, a single strain of toxigenic *C. ulcerans* was isolated from a carnivorous bird, ural owl (*Strix uralensis*). The bacterium was also isolated from two individuals of Japanese shrew-mole (*Urotrichus talpoides*), a food preference of the owl. Analysis by ribotyping showed that the owl and mole isolates were classified in a group, suggesting that *C*. *ulcerans* can be transmissible among wild birds and their prey animals. Moreover, our isolates were found to belong to a group of previously reported *C. ulcerans* isolates from dogs and a cat, which are known to serve as sources for human infection.

**Conclusion:**

The findings suggest that the shrew-mole may be a potential reservoir of a zoonotic pathogen *C. ulcerans*.

## Background

The toxigenic *Corynebacterium ulcerans* is a causative agent of diphtheria-like illness in humans. Diphtheria has long been recognized to be exclusively caused by *Corynebacterium diphtheriae* which circulates mostly among humans [1, 2]. Recently, along with the emergence of toxigenic *C. ulcerans* infection, the latter has been incorporated into the category of “diphtheria” in regions including European Union [3]. The cases caused by the organism is becoming the majority of diphtheria-related diseases in industrialized countries [4]. Compared with the host range of *C. diphtheriae* that is limited to humans, *C. ulcerans* exploits a wide range of mammalian hosts.

Companion animals such as dogs [5–9] and cats [10–13], as well as pigs [14, 15] have been shown to harbor *C. ulcerans* and may play a role as sources of infection for human. However, whether cats or dogs are the natural hosts for the bacterium is still unknown. In fact, the host range of the bacterium is not limited to these companion animals. Cattle [16, 17], roe deer (*Capreolus capreolus*) [18], wild boar (*Sus scrofa*) [19], goat [20], killer whale (*Orcinus orca*) [21], lion (*Panthera leo*) [21], ferret (*Mustela putorius furo*) [22] and dromedary camel (*Camelus dromedarius*) [23], have been reported to be affected by the bacterium. Despite the wide host range, so far the transmission of the bacterium from wild animal to humans has not been reported, or may be extremely rare. Wild animals have not been reported to serve as reservoir for infection of humans or companion animals so far. However, due to possible close contact between companion and wild animals including stray or freely roaming ones, information on how the bacterium is maintained and circulated among wild animals may greatly contribute to the prevention of *C. ulcerans* infection in companion and domestic animals. In addition, information is lacking about the presence and infection of *C. ulcerans* in non-mammalian vertebrates, including birds that comprise a diverse range of wildlife and often in close contact with prey wild mammalian hosts.

In this study, we performed a survey of *C. ulcerans* among wild birds and animals in Japan. The results of the survey and findings concerning the feeding behavior are described.

## Results

### Isolation of *C. ulcerans*

**Fig. 1:**
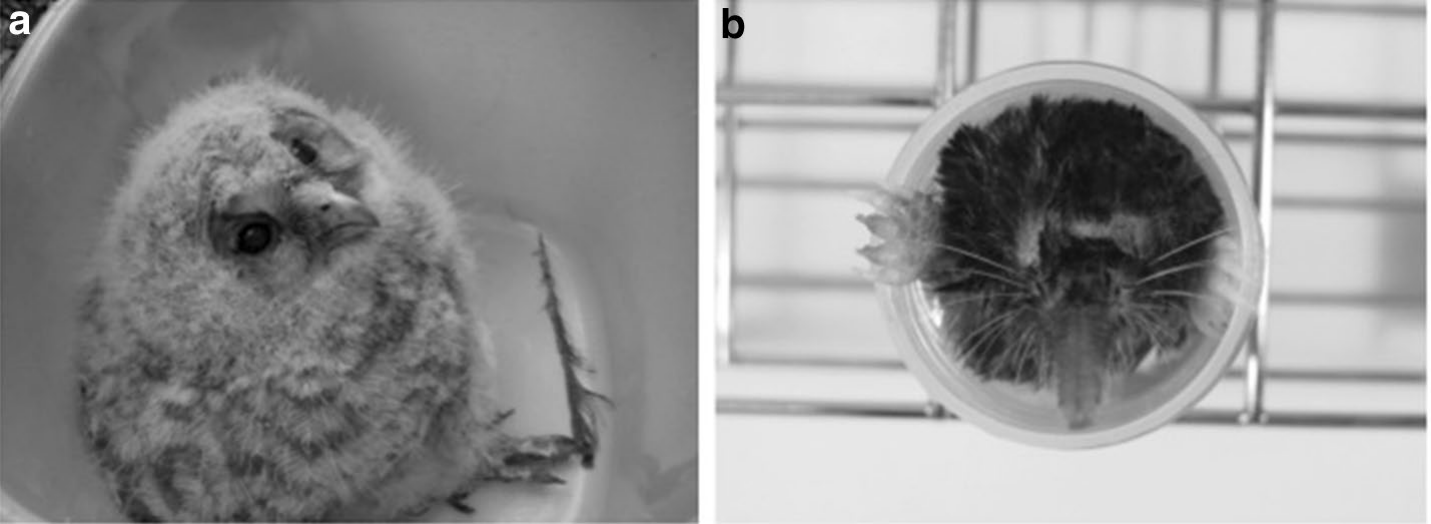
Ural owl and shrew-mole. **a** 4-week-old Ural owl (*Strix uralensis*) from which *C.* *ulcerans* was detected, found in its nest. **b** Male Japanese shrew-mole (*Urotrichus talpoides*) from which *C.* *ulcerans* was isolated, captured in a trap

We investigated the presence of toxigenic *C. ulcerans* in 380 wild birds (Table 1) and obtained one isolate (Owl1205) from only one young (4 weeks of age) Ural owl (*Strix uralensis*) captured from a nest (Fig. 1a). The sex of the owl was not established. It looked healthy and did not exhibit any clinical signs of illness. In the nest, investigation revealed that there were leftovers which comprised 18 mice of unknown species, 7 Japanese shrew-moles (*Urotrichus talpoides*), one Japanese mole (*Mogera wogura*) and birds and frogs of unknown species. In view of the possibility that the bacterium derived from the diet of the owl, we then tried to screen several small animals inhabiting the area surrounding the nest. Thirty-six small animals were captured, including 33 large Japanese field mice (*Apodemus speciosus*, 18 males and 15 females), 1 house mouse (*Mus musculus*, male), and 2 Japanese shrew-moles (one female and one male) (Fig. 1b). *C. ulcerans* was isolated from both the Japanese shrew-moles (strains Mole1212-1 and Mole1212-2) but was not detected in the other 34 animals (2 species of mice). The two shrew-moles did not exhibit any clinical signs of illness.

**Table 1:** List of captured birds

Family	Species	Number of inspections
Podicipedidae	*Tachybaptus ruficollis*	2
	*Podiceps cristatus*	1
Ardeidae	*Egretta garzetta*	2
	*Ardea cinerea*	2
Anatidae	*Cygnus olor*	1
Procellariidae	*Calonectris leucomelas*	3
Accipitridae	*Accipiter gentilis*	1
	*Accipiter nisus*	3
	*Butastur indicus*	1
	*Buteo japonicus*	2
Falconidae	*Falco peregrinus*	1
	*Falco tinnunculus*	2
Charadriidae	*Charadrius alexandrinus*	2
	*Vanellus cinereus*	2
Laridae	*Larus crassirostris*	1
	*Sterna albifrons*	20
	*Sterna dougallii*	7
Columbidae	*Columba livia*	35
	*Streptopelia orientalis*	8
Cuculidae	*Cuculus poliocephalus*	1
	*Cuculus saturatus*	1
Strigidae	*Strix uralensis*	13
Caprimulgidae	*Caprimulgus indicus*	3
Picidae	*Dendrocopos kizuki*	11
Passeridae	*Passer montanus*	17
Hirundinidae	*Hirundo rustica*	11
Pycnonotidae	*Hypsipetes amaurotis*	18
Turdidae	*Tarsiger cyanurus*	22
	*Turdus pallidus*	14
	*Turdus cardis*	4
	*Turdus chrysolaus*	1
Cettiidae	*Horornis diphone*	12
	*Urosphena squameiceps*	2
	*Phylloscopus borealoides*	2
	*Phylloscopus borealis*	5
Muscicapidae	*Cyanoptila cyanomelana*	2
	*Ficedula narcissina*	20
	*Muscicapa dauurica*	3
Paridae	*Parus varius*	15
	*Parus minor*	14
Zosteropidae	*Zosterops japonicus*	67
Emberizidae	*Emberiza cioides*	2
	*Emberiza spodocephala*	10
Fringillidae	*Carduelis sinica*	2
	*Uragus sibiricus*	1
Sturnidae	*Sturnus cineraceus*	2
Campephagidae	*Pericrocotus divaricatus*	2
Aegithalidae	*Aegithalos caudatus*	1
Corvidae	*Corvus corone*	3
	*Corvus macrorhynchos*	3
Total		380

### Characterization of isolates

The three *C. ulcerans* isolates obtained from the bird and animals showed the same API code (0111326,  % id 99.7, T = 1.0). The isolates were tested for toxigenicity and genetically analyzed. Using the Elek test (in-gel immunodiffusion test) and PCR for the toxin gene indicated that the three isolates were toxigenic.

**Fig. 2:**
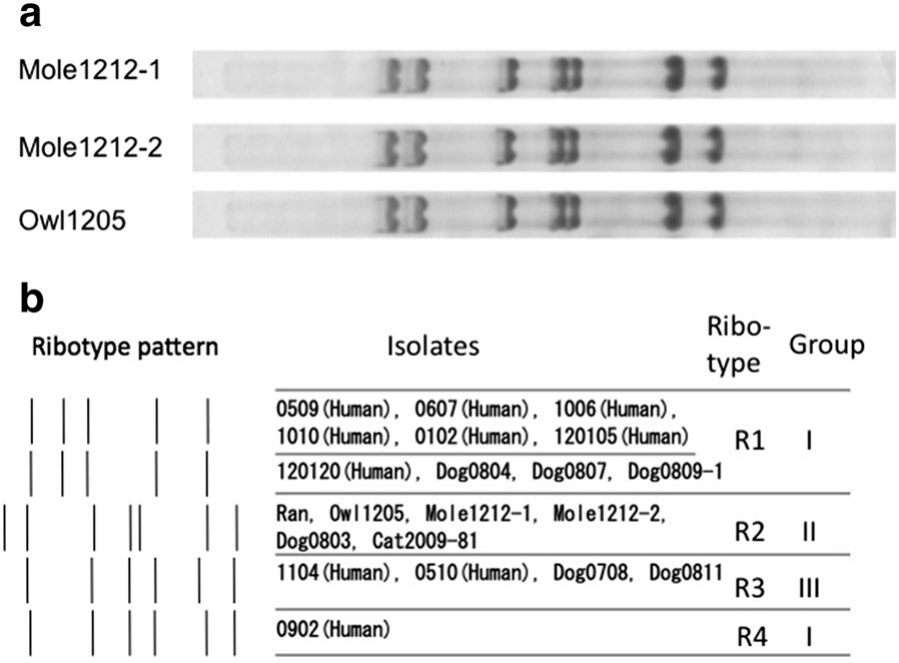
Ribotyping. **a** Ribotyping patterns of the owl and shrew-mole isolates. **b** Schematic view of ribotype patterns of the owl and shrew-mole isolates, displayed together with the patterns of other *C. ulcerans* isolate. The classifications of the isolates [24] are indicated on the right side of the figure

So far, *C. ulcerans* isolated in Japan have been categorized into 4 types by ribotyping (Fig. 2b) [24]. The owl and shrew-mole isolates showed identical ribotypes (Fig. 2a). Other ribotypes (R1, R3 and R4) represented human and companion animal isolates. Together with PFGE and toxin gene sequence the isolates have been categorized into three groups [24]. Owl, shlew-mole, killer whale and outdoor-bred dog were categorized together in one group (Group II) that did not contain human isloates.

The owl and shrew-mole isolates were susceptible to the antibiotics PCG, ABPC, CEX, CFPM, CTX, CTRX, IPM, VCM, GM, EM, CPFX, TC, ST, RFP, QPR/DPR, and LZD. The minimal inhibitory concentration of CLDM was 2 µg/ml in these strains and were considered intermediate. These results were consistent with the results of susceptibility tests on Canadian *C. ulcerans* isolates [25].

## Discussion

*Corynebacterium ulcerans* infection has been recognized as a zoonotic disease [26] and several kinds of wild animals, in addition to companion animals and livestock [5–24], are reported to be affected by the bacterium. However, information on the prevalence of the species among wild birds has been lacking.

In this study, we investigated 380 wild birds and isolated toxigenic *C. ulcerans* from a young owl probably fed by its parents. Then we continued our investigation on wild small rodents potentially serving as prey for owls and other animals.

A toxigenic bacterium of the same ribotype was isolated from two shrew-moles that constitute a major diet for the ural owl. This finding suggests that *C. ulcerans* could be transmitted from animals to birds through the food chain. Interestingly, in this study, *C. ulcerans* was not detected in mice, many more individuals of which were captured during the study. *C. ulcerans* was not detected from other bird species investigated. This may be explained by the feeding behavior of the birds. The owl is classified as raptors and is carnivorous, while the majority of other birds (i.e. except 22 raptors) eat plants, insects, larvae, fish, or aquatic organisms. If shrew-moles are natural hosts for *C. ulcerans*, the possibility of transmitting the bacteria from shrew-moles to non-carnivorous birds would be low.

By ribotyping (Fig. 2b; R2), PFGE and the sequence of the *tox* gene [24], the owl and mole isolates were found to form a group along with isolates from some other wild and free-roaming animals. The group consists of a killer whale isolate (Ran) [21], an isolate from a hunting dog [24] an isolate from a dog bred in a riverbed by a homeless person (dog0803) [6] and an isolate from a free-roaming cat (Cat2009-81) [24].

The other isolates were categorized into two groups (Fig. 2, Groups I and III), both of which contained human and companion animal isolates. All analytical methods employed in this study resulted in the same grouping, except that the group I was divided into two ribotypes (Fig. 2b, R1 and R4) [24].

So far, the present data suggest that the major part of circulating group II isolates is limited within wildlife and that the group is not likely to be an immediate threat to humans. However, the isolation of group II bacteria from a hunting dog (dog0907) [24], an outdoor-bred dog (dog0803) and a free-roaming cat suggests that cross infection between the wildlife and domestic animals may occur. Once a dog acquires *C. ulcerans*, the bacterium can be spread to other dogs [6]. Dogs are reportedly shown or suspected to be able to serve as sources for human infection, when placed in close contact with humans [7, 27, 28].

In this context, further investigation into the prevalence of *C. ulcerans* among wild animals will provide useful information on the source of *C. ulcerans*, track the potential source for human exposure, or at least, on revealing how the bacterium circulates in the natural environment.

## Conclusions

We isolated toxigenic *Corynebacterium ulcerans*, a zoonotic pathogen, from ural owl and two Japanese shrew-moles, their prey animals. The isolates formed a single (group II) cluster with isolates from a hunting dog, an outdoor-bred dog and a free-roaming cat. Prey animals potentially serve as source of *C. ulcerans* infection in carnivorous birds, dogs and cats, possibly leading to human infection.

## Methods

### Sample collection from birds

Between May 2011 and March 2013, throat swabs were collected from 380 wild birds (Table 1). The birds were captured for protecting them from illness or injury, for bird banding using mist net, or by hand. The captured birds were released after minimal handling and sample collection. Subjects also included birds temporarily captured for several reasons. Throat swabs were stored in modified Amies preservation medium [SEEDSWAB γ (gamma) 2; “Eiken” (Eiken Chemical, Tokyo, Japan)] at room temperature. Sample collection from birds was carried out under permission of national administration in charge of wild life and environment (Kinki Regional Office of the Ministry of Environment of Japan). The bird part of this study included only sampling from birds captured in the wild environment and was not subject for institutional ethics committee. The handling of birds corresponded to SCAW category B (experiments on vertebrate animal species that are expected to produce little or no discomfort) [29]. All of the birds were released after sampling.

### Sample collection from small animals

Between December 2012 and March 2013, throat swabs were collected from 36 small wild animals captured by a trap (H. B. Sherman Traps, Inc., Tallahassee, Florida, USA). Swabs were stored in the same way as those from birds. The animals were identified to the species level by observing their external anatomical morphology and by determining the DNA sequence of the D-loop non-coding region of mitochondrial DNA isolated from their hair [30]. Primers used for sequence determination were 5′-TCCCCACCATCAGCACCCAAAGC (forward) and 5′-TGGGCGGGTTGTTGGTTTCACGG (reverse). Sample collection from small animals was carried out under permission of prefectural administration in charge of wild life and environment (Chubu Office for Agriculture and Green of Osaka Prefectural Government). The animal part of this study included only sampling from animals captured in the wild environment and was not subject for institutional ethics committee. The handling of small animals corresponded to SCAW category B (experiments on vertebrate animal species that are expected to produce little or no discomfort) [29]. All of the animals were released after sampling.

### Bacterial isolation and identification

Each specimen was inoculated onto sheep blood agar and Katsukawa medium [charcoal–tellurite blood agar containing heart infusion agar, 0.03 % (w/v) potassium tellurite, 10 % (v/v) sheep blood and 0.05 % activated charcoal, hereafter referred as K medium] [5, 6] and was incubated at 35 °C. Colonies suspected to represent *C. ulcerans* that appeared after 18–24 h on sheep blood agar and 24, 30 and 48 h on K medium were transferred to dextrose-stärke-saccharose agar medium [31] to evaluate glucose and sucrose fermentation. The isolates that were positive for glucose but negative for sucrose fermentation were then characterized by Gram staining, catalase and urease tests. Gram-positive as well as catalase- and urease-positive organisms were suspected to be *C. ulcerans* and subjected to further identification using API Coryne (SYSMEX bioMérieux, Tokyo, Japan) kit, followed by the determination of partial RNA polymerase β-subunit (*rpoB*) gene sequences [32].

### Bacterial isolates and strains

*Corynebacterium ulcerans* isolates Owl1205, Mole1212-1 and Mole1212-2 were obtained in this study. Other strains are listed elsewhere [24]. These include novel isolates from wild animals, strains isolated from human and killer whale and from dogs during a survey in Osaka Prefecture.

### Toxigenicity testing and nucleotide sequencing of *tox* gene

Toxigenicity testing was performed by modified Elek test, Vero cell cytotoxicity and neutralization tests [6]. Polymerase chain reaction (PCR) targeting the gene for the A subunit of the diphtheria toxin (*tox*) [33] was used for screening the *tox* gene. For PCR-positive isolates, the entire *tox* gene was amplified with the primers toxFw and toxRv [21], and the nucleotide sequence of the amplified fragment was determined with primers placed at appropriate intervals. Tox gene sequences have been deposited to GenBank [Acc. No. AB926012.1 (Owl1205), AB926013.1 (Mole1212-1) and AB926014.1 (Mole1212-2)].

### Ribotyping

Ribotyping of bacterial strains was performed as described by De Zoysa et al. [34] and Regnault et al. [35]. Genomic DNA from *C. ulcerans* was digested with *Bst*EII (Roche Diagnostics), electrophoresed in an agarose gel, and transferred to HyBond Plus nylon membrane (Amersham Biosciences (GE Healthcare), Tokyo, Japan). The transferred DNA was then hybridized with a DIG-labeled OligoMix 5 probe mixture [36], and signals were detected with an alkaline phosphatase-conjugated anti-DIG antibody (Roche Diagnostics).

### Antibiotic susceptibility

Antibiotic susceptibility tests were performed by the broth microdilution method using Dry Plate (Eiken Chemical) for benzylpenicillin (PCG), ampicillin (ABPC), cephalexin (CEX), cefepime (CFPM), cefotaxime (CTX), cefriaxione (CTRX), imipenem (IPM), vancomycin (VCM), gentamicin (GM), erythromycin (EM), ciprofloxacin (CPFX), tetracycline (TC), clindamycin (CLDM), sulfamethoxazole–trimethoprim (ST), rifampicin (RFP), quinupristin–dalfopristin (QPR/DPR), and linezolid (LZD). Sensitivities were assessed according to the Clinical and Laboratory Standards Institute (CLSI) standard criteria (M45-A) for *Corynebacterium* species. For two drugs, for which criteria are lacking, standards for similar drugs were applied (ABPC for PCG and CEX for CTX).
